# Epidemiology, clinical features and outcome of adults with meningococcal meningitis: a 15-year prospective nationwide cohort study

**DOI:** 10.1016/j.lanepe.2023.100640

**Published:** 2023-04-28

**Authors:** Thijs M. van Soest, Nora Chekrouni, Nina M. van Sorge, Merijn W. Bijlsma, Matthijs C. Brouwer, Diederik van de Beek

**Affiliations:** a Department of Neurology, Amsterdam Neuroscience, Amsterdam UMC, Location University of Amsterdam, Meibergdreef, Amsterdam, the Netherlands; b Department of Medical Microbiology and Infection Prevention, Amsterdam UMC Location University of Amsterdam, Amsterdam Institute for Infection and Immunity, Meibergdreef, Amsterdam, the Netherlands; c Netherlands Reference Laboratory for Bacterial Meningitis, Amsterdam UMC Location AMC, Amsterdam, the Netherlands; d Department of Paediatrics, Amsterdam Neuroscience, Amsterdam UMC Location University of Amsterdam, Meibergdreef, Amsterdam, the Netherlands

**Keywords:** Meningococcal meningitis, Epidemiology, Clinical features, Outcome

## Abstract

**Background:**

We describe the epidemiology, clinical features and outcome of adult meningococcal meningitis in the Netherlands over a 15-year period.

**Methods:**

We studied adults (age ≥ 16 years) who were listed by the Netherlands Reference Laboratory for Bacterial Meningitis and/or included in the prospective nationwide cohort study (MeninGene) between January 2006 and July 2021. Incidences were calculated per epidemiological year (July–June).

**Findings:**

We identified 442 episodes of adult meningococcal meningitis. The median patient age was 32 years (IQR 18–55) and 226 episodes (51%) occurred in female patients. The annual incidence per 100,000 adults fluctuated, from 0.33 in 2006–2007 to 0.05 in 2020–2021, with a temporal increase up to 0.30 from 2016 to 2018, driven by an outbreak of serogroup W (MenW). Of 442 episodes, 274 episodes (62%) in 273 patients were included in the clinical cohort study. The overall case fatality rate was 4% (10 of 274) and 16% (43 of 274) had an unfavourable outcome (Glasgow Outcome Scale score 1–4). Compared to other serogroups, MenW was associated with higher rates of unfavourable outcome (6 of 16 [38%] *vs.* 37 of 251 [15%], P = 0.03) and death (4 of 16 [25%] *vs*. 6 of 251 [2%], P = 0.001).

**Interpretation:**

The overall incidence of adult meningococcal meningitis in the Netherlands is low and outcome is generally favourable. An increase of MenW meningitis occurred from 2016 to 2018, which was associated with more unfavourable outcome and death.

**Funding:**

10.13039/501100001826Netherlands Organisation for Health Research and Development, 10.13039/501100000781European Research Council, National Institute of Public Health and Environmental protection.

**Research in context**

**Evidence before this study**

We searched Pubmed for prospective cohort studies on epidemiological data, clinical characteristics or outcome of meningococcal meningitis in adults, combining the Mesh term “Meningitis, meningococcal” and “adults”, with “epidemiology” or “clinical characteristics” or “outcome”. Worldwide, introduction of vaccines following outbreaks of serogroup C (MenC vaccine) around the turn of the millennium, and more recently MenW and MenY (MenACWY vaccine), have reduced incidence rates and altered serogroup distribution. Several higher resource countries, but not the Netherlands, started routine immunisation against MenB (4CMenB vaccine). In previous clinical studies on meningococcal meningitis in adults, mortality rates were low compared to other common causes of bacterial meningitis. Age, sepsis-related features and the MenC strain of clonal complex 11 have been associated with unfavourable outcome in cohorts of patients with meningococcal meningitis. MenW strains of clonal complex 11 have also been associated with increased disease severity and death upon invasive meningococcal disease.

**Added value of this study**

Our study describes an overview of adult meningococcal meningitis over the last 15 years by analysing the epidemiology, clinical characteristics, outcome, and prognostic factors of meningococcal meningitis in adults in the Netherlands. Serogroup B was the most common serogroup (77%), and the 4CMenB vaccine was predicted to potentially prevent 71% of adult meningococcal serogroup B meningitis episodes. An increased incidence occurred between 2016 and 2018, driven by a hyperendemic period of MenW, mostly belonging to clonal complex 11. MenW disease was associated with features of sepsis, systemic complications and unfavourable outcome, compared to all other serogroups. The incidence of MenW meningitis declined after implementation of the conjugate vaccine against serogroups A, C, W and Y.

**Implications of all the available evidence**

This study highlights the need for continuous monitoring of epidemiological and clinical data, such as capsule switching of the clonal complex 11, which may require changes in immunisation programmes across the world**.** Conjugate vaccines against several meningococcal serogroups have been successful in reducing the incidence of meningococcal meningitis. Vaccination against MenB may be required in the future due to epidemiological changes. When this happens, the 4CMenB vaccine has the potential to be included in the Dutch national immunisation programs.

## Introduction

*Neisseria meningitidis* is a Gram-negative bacterium that can cause severe infections, including sepsis and meningitis.^1^^,^^2^ This bacterium causes 10–15% of community-acquired meningitis cases in adults, of which most cases occur in younger adults.^3^^,^^4^ The case fatality rate has been reported to be 3–4%.^3^^,^^5^ The meningococcal polysaccharide capsule is used to classify the bacterium in multiple clinically-relevant serogroups (A, B, C, W, X, Y, Z).^6^^,^^7^ Beyond serogrouping, meningococci can be further classified in clonal complexes based on the specific sequence of seven housekeeping genes. The hypervirulent clonal complex 11 (cc11) strain has caused several outbreaks in the past,^2^ and has undergone different capsule switching events e.g. from serogroup C to serogroup W.^8^^,^^9^

The incidence of meningococcal meningitis and relative distribution of serogroups can vary widely by region and over time, and has been impacted by the introduction of meningococcal conjugate vaccines worldwide.^6^^,^^7^ In the Netherlands, routine MenC vaccination at 14 months was implemented in 2002 following an increase in MenC cases.^10^ Simultaneously, children aged 1–18 years were offered a single dose in a catch-up campaign. Besides a decline in incidence in vaccinated children, a large decline was also observed in the unvaccinated population due to reduced carriage and transmission of meningococci (herd protection).^10^^,^^11^ After an increase in MenW cases in the Netherlands in the period 2015–2017, the MenC conjugate vaccine was replaced by the tetravalent conjugate MenACWY vaccine in 2018.^12^ This vaccine similarly decreased carriage and disease rates of the four serogroups.^13^ For serogroup B, the polysaccharide capsule is poorly immunogenic and unsuitable as a vaccine antigen. To prevent MenB disease, two protein-based vaccines are available. The 4CMenB (Bexsero®) vaccine based on three highly immunogenic antigens (fHbp, NadA, and NHBA), and the outer membrane vesicle from a New Zealand MenB outbreak strain (B:4:P1.7–2,4). This vaccine is registered for use in adults and children and has been implemented in the infant immunisation programme of multiple countries but not in the Netherlands.^14^ The other available vaccine (MenB-FHbp, Trumenba®) is based on two variants of the fHbp and has been registered for persons of 10 years of age and older.^15^ As these vaccines target antigens that are also present in other serogroups, they can also offer cross-protection against other serogroups.^16^^,^^17^

We previously described outcome of adults with meningococcal meningitis included in cohorts from 1998–2002 and 2006–2010 to evaluate the implementation and effectiveness of adjunctive dexamethasone in meningococcal meningitis.^4^ Since 2006, we perform a prospective nationwide cohort study on community-acquired bacterial meningitis in adults (MeninGene).^3^^,^^18^ Over the past 15 years, an outbreak of MenW has occurred and new conjugate vaccines have been introduced in the Netherlands. Here, we report on the epidemiology, clinical features, outcome and prognostic factors of adult community-acquired meningococcal meningitis in the Netherlands over a 15-year period.

## Methods

We included adults (aged 16 years or older) with meningococcal meningitis in the Netherlands between January 1st, 2006 and July 1st, 2021. We combined national bacteriological surveillance data of the Netherlands Reference Laboratory for Bacterial Meningitis (NRLBM) and episodes identified by the MeninGene cohort study, a nationwide study of community-acquired bacterial meningitis in the Netherlands.^3^^,^^18^ The NRLBM receives approximately 90% of cerebrospinal fluid (CSF) isolates and samples of bacterial meningitis patients from clinical microbiology laboratories throughout the Netherlands.^19^ The MeninGene study prospectively includes patients that are either notified to the researchers by their treating physician or listed by the NRLBM. The NRLBM provided the researchers with a daily report including name of the hospital and treating physician.^19^ Up to 2019, only CSF-positive cultures were reported, afterwards also culture-negative CSF samples with positive polymerase chain reaction (PCR). The researchers contacted the treating physician and provided information about the study. All patients, or their legal representatives, were given written information and were asked to provide written informed consent to allow inclusion in the study. Clinical data, including presenting features, laboratory results, clinical course and outcome were collected using a case record form (CRF) for the patients included in the MeninGene study.

Meningococcal meningitis was defined as either having a positive CSF bacterial culture, PCR-positive CSF, CSF *N. meningitidis* antigen test*,* or a meningococcal positive blood culture combined with one or more individual predictors of bacterial meningitis defined by Spanos and colleagues.^20^ These predictors consist of CSF glucose concentration <1.9 mmol/L (340 mg/L), CSF:blood glucose ratio <0.23, protein concentration >2.2 g/L, white blood cell count >2000 per mm^3^ or >1180 polymorphonuclear leukocytes per mm^3^. All episodes fulfilling this criteria were included, also if sepsis and meningitis were both present.

Distant foci of infection were defined as otitis, sinusitis or endocarditis, and were only considered missing if all were missing. Neurological examination was recorded at admission and discharge. A Glasgow Coma Scale (GCS) score below 8 was defined as coma and a GCS score below 14 was defined as altered mental status. Outcome was scored on the Glasgow Outcome Scale (GOS), which ranges from 1 (death) to 5 (mild or no disability). An unfavourable outcome as defined as a GOS score of 1 through 4.

Meningococcal serogrouping was routinely performed by the NRLBM using Ouchterlony gel diffusion on isolates from CSF and blood, or by meningococcal-specific and group-specific real-time PCR, as described previously.^21^ As comparison, we included genome sequences (WGS) from multiple sources. One unpublished study sequenced approximately 50 meningococcal isolates from 2005 and 2010 each. Another previous study sequenced meningococcal isolates between 2006-2013.^22^ Finally, we included genome sequences from the routine WGS programme of the NRLBM, which started in August 2016. Typing of isolates was supplemented with Sanger sequencing of MLST genes as performed by the NRLBM up to 2011.

To predict coverage of meningococcal isolates by the 4CMenB vaccine, Bexsero® Antigen Sequence Typing (BAST) was performed and the Meningococcal Deduced Vaccine Antigen Reactivity (MenDeVAR) index was used.^23^^,^^24^ We considered isolates with an “exact match” or those who were “cross-reactive” to be covered by the vaccine.

Available isolates were tested for penicillin susceptibility by E-test to determine minimum inhibitory concentrations (MIC). Isolates were considered susceptible if MIC≤0.25 and resistant if MIC>0.25 (European Committee on Antimicrobial Susceptibility Testing [EUCAST] breakpoint).

The incidences of meningococcal meningitis were calculated per epidemiological year (1st of July – 30th of June) per 100,000 inhabitants of the Netherlands aged 16 years or older (data from CBS Statline), using all episodes registered by the NRLBM or included in the MeninGene study. Incidences were compared using the incidence rate ratio (IRR), using the “epitools” package (version 0.5–10.1) in R, reporting the IRR and 95% confidence interval (95% CI). Continuous variables were presented as medians with interquartile range (IQR) and categorical variables were presented as proportion of the episodes for which the data was known. We tested for differences between groups, using the Mann–Whitney U test for continuous variables, and Fisher's exact test for categorical variables. We performed a univariable logistic regression and chose the possible predictors based on previous research, pathophysiological interest and selected characteristics that were measured on admission. The association between the variables and unfavourable outcome was investigated, providing odds ratios (ORs) and 95% confidence intervals (95% CIs). Linearity of the association of continuous variables and unfavourable outcome was visually assessed. If no linear relationship was found, the variable was categorized. We chose the reference category as either the category including the most episodes, or the category that we considered the most interesting to compare to. All analyses were conducted in R version 4.0.3 and a p-value <0.05 was considered statistically significant.

The funders had no role in study design, data collection, data analysis, interpretation or writing of the report.

## Results

**Fig. 1:**
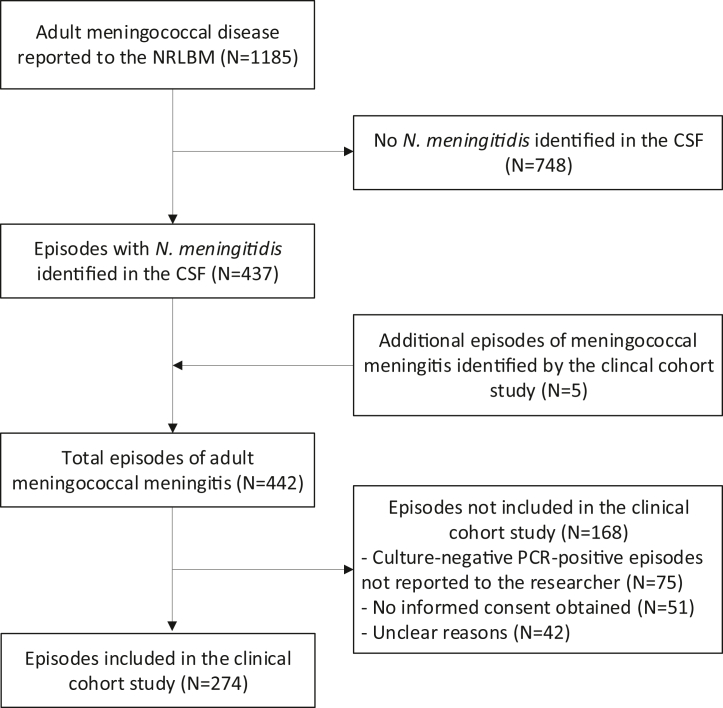
**Selection of episodes**. NRLBM = Netherlands Reference Laboratory for Bacterial Meningitis; CSF = cerebrospinal fluid.

**Fig. 2:**
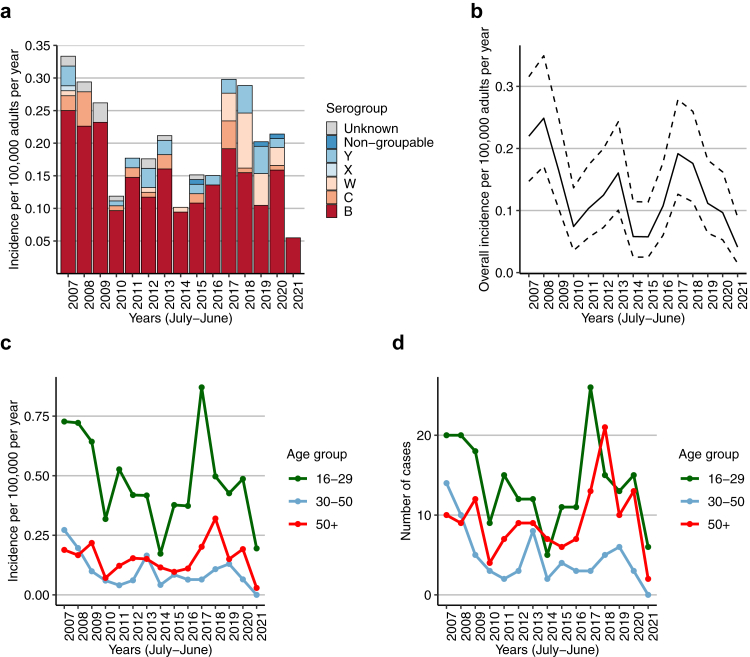
**Epidemiology of meningococcal meningitis**. (A) Stacked bar chart of the incidence of meningococcal meningitis per epidemiological year (July–June) per serogroup. (B) Incidence of meningococcal meningitis episodes included in the nationwide clinical cohort study (MeninGene), with the 95% confidence interval (dashed lines). (C) Incidence of meningococcal meningitis per age group per epidemiological year. (D) Number of cases per age group per epidemiological year.

In total, 1,185 episodes of adult meningococcal disease were reported to the NRLBM, of which 437 were meningitis. An additional 5 additional episodes were included in the MeninGene study, resulting in 442 included episodes (Fig. 1). *N. meningitidis* was detected in CSF in 432 episodes (98%): by positive culture in 308 (70%) and species-specific PCR in 124 (28%). Ten episodes (2%) were included with only a positive blood culture and at least one CSF indicator of bacterial meningitis. The incidence was variable (Fig. 2, panel A), but over the study period the overall incidence declined from 0.33 per 100,000 adults per year in 2006–2007 to 0.05 per 100,000 adults per year in 2020–2021 (IRR = 0.16 [95% CI 0.08–0.35], P < 0.001). In the period between 2016–2017 and 2017–2018, a temporal increased incidence occurred with annual incidences of 0.30 per 100,000 adults in 2016–2017 and 0.29 per 100,000 adults in 2017–2018 (Fig. 2).

### Patient characteristics and clinical features

The median age was 32 years (IQR 18–55) and 226 of 442 episodes (51%) occurred in female patients. The annual incidence was highest in patients aged 16–29 years, ranging from 0.17 to 0.87 per 100,000 (Fig. 2, panel C). After a declining incidence between 2007–2014, the incidence in this age group increased from 0.17 to 0.87 per year in 2016–2017 (IRR 5.04 [95% CI 1.93–13.12], P < 0.001), and subsequently declined after 2018. In patients older than 50 years of age, the annual incidence increased from 0.10 in 2014–2015 to 0.32 in 2017–2018 per 100,000 adults (IRR 3.69 [95% CI 1.49–9.15], P = 0.002), and subsequently declined. This age group also had the highest number of episodes in 2018 (n = 21, Fig. 2, panel D).

Of 442 episodes, 274 episodes (62%) in 273 patients were included in the prospective nationwide cohort study (MeninGene; Fig. 1). One patient was included twice with two different episodes. Of the 168 episodes that were not included, 75 (45%) were culture-negative PCR positive and were not reported to the investigators up to 2019, in 51 episodes (30%) the patient was identified but no informed consent was obtained because permission of hospitals was still pending, or the patient or physician refused participation, and in the remaining 42 episodes (25%) the reasons for failed inclusion were unclear. In 36 of 274 episodes (13%), the patient had immunocompromising conditions, consisting of a medical history including diabetes mellitus, infection with the human immunodeficiency virus, use of immunosuppressant drugs, alcoholism or active cancer (Table 1). Distant infection foci were identified on admission in 20 of 270 episodes (7%) and consisted of pneumonia in 7 of 266 (3%) and otitis or sinusitis in 13 of 259 (5%). Patients included in the nationwide cohort study had a comparable age and sex to those not included (33 [IQR 19–55] vs 29 years [IQR 18–57], P = 0.20; 142 females in 274 episodes [52%] vs 84 females in 168 episodes [51%], P = 0.84).

**Table 1:** Baseline characteristics of patients included in the nationwide cohort study.

Characteristic	
**Demographics**	
Age (years)^a^	33 (19–55)
Female sex	142/274 (52%)
Immunocompromised	36/274 (13%)
Diabetes Mellitus	21/270 (8%)
HIV	3/273 (1%)
Immunosuppressive drugs	5/272 (2%)
Alcoholism	8/274 (3%)
Active cancer	1/273 (0.3%)
Extrameningeal infection	20/270 (7%)
Pneumonia	7/266 (3%)
Otitis or sinusitis	13/259 (5%)
Antibiotic pre-treatment	8/265 (3%)
Duration of symptoms <24 h	128/265 (48%)
**Symptoms on admission**	
Headache	230/251 (92%)
Nausea	174/240 (72%)
Neck stiffness	191/251 (76%)
Rash	119/258 (46%)
Temperature (degrees Celsius)^b^	38.1 (37.1–39.0)
Fever (≥38.0 °C)	141/265 (53%)
Heart rate (beats/minute)^c^	90 (80–105)
Systolic blood pressure (mmHg)^d^	126 (110–141)
Diastolic blood pressure (mmHg)^e^	72 (62–82)
GCS score on admission^f^	14 (11–15)
Altered mental status (<14)	122/273 (45%)
Coma (<8)	23/273 (8%)
Seizures	8/264 (3%)
Cranial nerve palsy	6/241 (2%)
Mono-, hemiparesis or aphasia	28/259 (11%)
Triad	54/263 (21%)
**Blood results**	
Thrombocytes (x10ˆ9/L)^g^	182 (144–234)
Leukocytes (x10ˆ9/L)^h^	18.9 (14.4–23.3)
C-reactive protein (mg/L)^i^	223 (135–312)
Positive blood culture	121/227 (53%)
**CSF results**	
Leukocytes (per mm^3^)^j^	5730 (1820–12,225)
**<**100 (per mm^3^)	18/254 (7%)
100-999 (per mm^3^)	20/254 (8%)
>999 (per mm^3^)	216/254 (85%)
CSF:blood glucose ratio^k^	0.10 (0.01–0.32)
Protein (g/L)^l^	3.91 (1.97–6.28)
Opening pressure (cm H20)^m^	41 (30–50)
Positive Gram stain	162/213 (76%)
Positive CSF culture	231/274 (84%)

Data as n/N (%) or median (IQR).

a Age known in 274 episodes.

b Temperature known in 265 episodes.

c Heart rate known in 262 episodes.

d Systolic blood pressure known in 264 episodes.

e Diastolic blood pressure known in 264 episodes.

f GCS score known in 273 episodes.

g Blood thrombocyte count known in 255 episodes.

h Blood leukocyte count known in 268 episodes.

i Blood CRP known in 259 episodes.

j CSF leukocyte count known in 254 episodes.

k CSF:blood glucose ratio known in 246 episodes.

l CSF protein known in 258 episodes.

m Opening pressure known in 113 episodes.

Common presenting features were headache, occurring in 230 of 251 episodes (92%), and neck stiffness (191 of 251 [76%]; Table 1). Fever on admission was present in 141 of 265 episodes (53%) and rash in 119 of 258 (46%), being petechial in 96 of these episodes (81%). Focal cerebral deficits (aphasia, mono- or hemiparesis) occurred in 28 of 259 episodes (11%) and cranial nerve palsies in 6 of 241 episodes (2%). The triad of fever, neck stiffness and an altered consciousness was present in 54 of 263 episodes (21%). A lumbar puncture was performed in all included patients. The median CSF leukocyte count was 5730 per mm^3^ (IQR 1820–12,225) and CSF Gram staining showed bacteria in 162 of 213 episodes (76%). CSF cultures were positive in 231 of 274 (84%) and CSF PCR was performed and positive in 46 of 274 (17%). Blood cultures were positive in 121 of 227 episodes (53%). Out of 153 episodes with cranial imaging on admission, it was performed before lumbar puncture in 123 of 137 (90%). Antibiotics were started prior to cranial imaging in 39 of 120 (33%) of these episodes. Antibiotic treatment was started prior to cranial imaging in 23 of 33 episodes (70%) with negative CSF cultures, compared to 36 of 101 episodes (36%) with positive CSF cultures and cranial imaging performed (P = 0.001).

### Meningococcal serogroup, clonal complex and antibiotic susceptibility

Serogroup data were available for 428 episodes (97%), of which 328 episodes were caused by serogroup B (77%; Supplementary Table S1), 35 by serogroup Y (8%), 33 by serogroup W (8%), 27 by serogroup C (6%), 2 by serogroup X (0.5%) and in 3 episodes the meningococci were non-groupable (0.7%). In 4 of 27 MenC episodes (15%), the patient had been eligible for vaccination since 2002. Twenty-nine of 33 episodes (88%) of MenW meningitis occurred between 2016 and 2020. After implementation of MenACWY in May 2018, 13 episodes of MenW and 10 episodes of MenY meningitis occurred, of which none occurred in patients who had been eligible for vaccination. No more episodes caused by these serogroups occurred after February 2020. 4CMenB reactivity was determined for 154 MenB isolates, of which 109 (71%) were predicted to be covered by the vaccine: 88 (57%) were an exact match and 21 (14%) were cross-reactive. Fourteen (11%) were predicted not to be covered and 31 had insufficient data (20%). Additionally, 3 of 14 MenC isolates (21%; all cross-reactive), 2 of 19 MenY isolates (11%; both exact match) and 13 of 18 MenW isolates (72%; all cross-reactive) were predicted to be covered by the 4CMenB vaccine.

Out of 442 episodes, 328 meningococcal isolates (74%) were collected by the NRLBM, of which sequence types were determined in 237 (72%; missing sequence data were mostly from 2014–2016 [Supplementary Fig. S1]). This resulted in 106 unique sequence types. The most common clonal complexes were cc41/44 (66 [28%]), cc32 (57 [24%]) and cc11 (23 [10%]); Supplementary Table S1). Out of these 23 isolates belonging to cc11, 14 (61%) were MenW and 9 (39%) were MenC. Before 2011–2012, all cc11 isolates (7 isolates) were MenC and after 2011–2012, 14 of 16 (88%) were MenW. Other clonal complexes belonged mostly to one serogroup (Supplementary Table S1). Serogroups and clonal complexes did not differ between episodes which were included in the prospective cohort study and those which were not included (Supplementary Table S2). Penicillin susceptibility was tested for all 328 available isolates (100%), of which 327 (98%) were susceptible to penicillin (MIC ≤0.25), and 1 with MenW:cc11 meningitis (0.3%) was resistant (MIC >0.25).

### Treatment, complications and outcome

Initial antibiotic treatment consisted of a third-generation cephalosporin in combination with amoxicillin in 137 of 271 episodes (51%), of monotherapy with a third-generation cephalosporin in 76 (28%), of monotherapy with amoxicillin or penicillin in 45 (17%), and of another antibiotic regimen in 13 (5%). Adjunctive dexamethasone was started in 250 of 268 episodes (93%), and the first dose was given before or together with the initial antibiotics in 244 of 268 episodes (91%). It was continued for four days with a dosage of 40 mg per day in 234 of 253 episodes (92%).

Neurological complications, consisting of seizures, cerebral infarction, hydrocephalus, sinus thrombosis, and brain abscesses, occurred during admission in 19 of 266 episodes (7%; Table 2). Four patients (2%) developed cerebral abscesses. Focal neurologic deficits occurred during admission in 33 of 259 patients (13%). Systemic complications, including respiratory failure, pneumonia, circulatory shock and arthritis, occurred in 51 of 270 episodes (19%).

**Table 2:** Clinical course and outcome.

Characteristic	
**Complications**	
Pneumonia	9/258 (3%)
Arthritis	10/263 (4%)
Circulatory shock	23/262 (9%)
Respiratory failure	24/268 (9%)
Mechanical ventilation	43/268 (16%)
Cerebral infarction	6/260 (2%)
Hydrocephalus	4/257 (2%)
Sinus thrombosis	1/255 (0%)
Brain abscess	4/71 (6%)
Seizures	6/259 (2%)
**Glasgow outcome scale score**	
1 (death)	10/274 (4%)
2 (vegetative state)	0/274 (0%)
3 (severe disability)	6/274 (2%)
4 (moderate disability)	27/274 (10%)
5 (mild or no disability)	231/274 (84%)
**Neurologic sequelae**	
Cognitive impairment	30/220 (14%)
Hearing impairment	34/243 (14%)
Cranial nerve palsy	15/227 (7%)
Aphasia, mono- or hemiparesis	3/230 (1%)

Data presented as n/N (%).

Outcome was unfavourable in 43 of 274 episodes (16%) and 10 patients died (4%). The cause of death was sepsis or multi-organ failure in 6 of these patients (60%). The other patients died after withdrawal of care due to a poor neurological prognosis (n = 2), multiple cerebral infarctions (n = 1) and brain herniation (n = 1). Out of 214 episodes in which the patient survived, neurological sequelae including cognitive impairment, hearing impairment, cranial nerve palsies, aphasia, mono- or hemiparesis, occurred in 67 patients (31%). In a univariable analysis (Table 3), admission characteristics associated with an unfavourable outcome were increasing age, focal cerebral deficits, lower score on the Glasgow Coma Scale, higher heart rate, systolic blood pressure <90 mmHg (shock) a CSF leukocyte count <100 cells per mm^3^ and positive blood cultures. Low CSF leukocyte counts (<100 per mm^3^) were associated with lower systolic blood pressures, compared to CSF leukocytes of 1000–10000 per mm^3^ and CSF leukocytes> 10,000 per mm^3^, and lower serum thrombocytes compared to all other groups (100–999, 1000–10000, and >10,000 per mm^3^) in a univariable linear regression analysis (Supplementary Table S3). Furthermore, there was a trend towards more positive blood cultures compared to the other categories (83% vs 49–56%, P = 0.054 across all groups). C-reactive protein levels were overall lower in the group with CSF leukocytes <100 per mm^3^. The univariable odds ratio for unfavourable outcome and dexamethasone use was 0.52 (95% CI 0.22–1.31, P = 0.14).

**Table 3:** Characteristics associated with unfavourable outcome.

Characteristic	Favourable Outcome (N = 231)	Unfavourable outcome (N = 43)	Univariable odds ratio (95% CI)	P-value
Age	27 (19–52)	57 (41–70)	–	–
<30	122/231 (53%)	9/43 (21%)	0.29 (0.12–0.63)	0.003
30–70	98/231 (42%)	25/43 (58%)	*Reference*	*Reference*
>70	11/231 (5%)	9/43 (21%)	3.21 (1.18–8.62)	0.02
Pneumonia	5/224 (2%)	2/42 (5%)	2.19 (0.31–10.60)	0.36
Otitis or sinusitis	12/219 (5%)	1/40 (2%)	0.44 (0.02–2.34)	0.44
Immunocompromised state	27/231 (12%)	9/43 (21%)	2.00 (0.83–4.49)	0.10
Seizures	6/225 (3%)	2/39 (5%)	1.97 (0.28–8.94)	0.42
Headache	197/215 (92%)	33/36 (92%)	1.01 (0.32–4.46)	>0.99
Nausea	154/207 (74%)	20/33 (61%)	0.53 (0.25–1.16)	0.10
Neck stiffness	167/214 (78%)	24/37 (65%)	0.52 (0.25–1.12)	0.09
Rash	105/222 (47%)	14/36 (39%)	0.71 (0.34–1.44)	0.35
Fever (≥38.0 °C)	118/226 (52%)	23/39 (59%)	1.32 (0.66–2.66)	0.44
Systolic blood pressure <90 mmHg	4/225 (2%)	4/39 (10%)	6.31 (1.43–27.83)	0.01
Triad	43/224 (19%)	11/38 (29%)	1.71 (0.76–3.65)	0.17
Aphasia, mono- or hemiparesis	20/222 (9%)	8/37 (22%)	2.79 (1.07–6.73)	0.03
Cranial nerve palsy	4/209 (2%)	2/32 (6%)	3.42 (0.46–18.30)	0.17
Glasgow Coma Scale score^a^	14 (11–15)	12 (9–14)	0.85 (0.76–0.95)	0.004
Heart rate (beats/minute)^b^	90 (76–102)	100 (86–122)	1.38 (1.17–1.66)	<0.001
Blood C-reactive protein (mg/L)	230 (136–302)	194 (138–360)	–	–
<100	37/224 (17%)	5/35 (14%)	0.92 (0.29–2.46)	0.87
100–300	129/224 (58%)	19/35 (54%)	*Reference*	*Reference*
>300	58/224 (26%)	11/35 (31%)	1.29 (0.56–2.84)	0.54
CSF leukocytes (per mm^3^)	5730 (2170–12,499)	5582 (485–10,761)	–	–
<100	12/218 (6%)	6/36 (17%)	4.10 (1.27–12.30)	0.01
100–999	15/218 (7%)	5/36 (14%)	2.73 (0.80–8.26)	0.09
1000–10000	123/218 (56%)	15/36 (42%)	*Reference*	*Reference*
>10,000	68/218 (31%)	10/36 (28%)	1.21 (0.50–2.80)	0.67
CSF protein (g/L)	3.83 (1.95–6.10)	5.60 (3.27–7.02)	–	–
<1.00	25/222 (11%)	6/36 (17%)	1.50 (0.49–4.13)	0.44
1.00-3-99	91/222 (41%)	9/36 (25%)	0.62 (0.25–1.46)	0.28
4.00–9.00	94/222 (42%)	15/36 (42%)	*Reference*	*Reference*
>9.00	12/222 (5%)	6/36 (17%)	3.13 (0.97–9.45)	0.05
CSF:blood glucose ratio	0.10 (0.01–0.33)	0.08 (0.01–0.29)	–	–
<0.25	144/216 (67%)	20/30 (67%)	0.69 (0.23–2.57)	0.60
0.25–0.50	52/216 (24%)	6/30 (20%)	0.58 (0.15–2.45)	0.40
>0.50	20/216 (9%)	4/30 (13%)	*Reference*	*Reference*
Positive blood culture	97/192 (51%)	24/35 (69%)	2.14 (1.01–4.77)	0.05
Positive CSF culture	195/231 (84%)	36/43 (84%)	0.95 (0.41–2.47)	0.91
Serogroup			–	
B	179/224 (80%)	27/43 (63%)	*Reference*	*Reference*
C	11/224 (5%)	6/43 (14%)	3.62 (1.17–10.4)	0.019
W	10/224 (4%)	6/43 (14%)	3.98 (1.27–11.60)	0.01
Y	21/224 (9%)	4/43 (9%)	1.26 (0.35–3.63)	0.69
Other	3/224 (11%)	0/43 (0%)	*NA*	0.99
Clonal complex 11	11/161 (7%)	6/31 (19%)	3.27 (1.05–9.44)	0.03
Dexamethasone^c^	202/229 (88%)	31/36 (86%)	0.52 (0.22–1.31)	0.14

Data presented as n/N (%) or median (IQR).

a Glasgow Coma Scale score was evaluated 231 with a favourable outcome and 42 patients with an unfavourable outcome, odds ratio per 1 point increase.

b Heart rate was evaluated in 223 patients with favourable outcome and 39 patients with unfavourable outcome, odds ratio per 10 beats/min increase.

c Dexamethasone started before or together with initial antibiotics and continued for 4 days with a dosage of 40 mg per day.

### Associations between microbiological and clinical characteristics

We also analysed the impact of meningococcal serogroup on clinical features and outcome (Supplementary Table S4). MenB and MenY episodes had the lowest rates of unfavourable outcome (27 of 206 [13%] and 4 of 25 [16%] respectively) and death (4 of 206 [2%] and 0 of 25 [0%] respectively). Compared to all other serogroups, MenC was associated with a higher patient age (46 [IQR 31–62] vs 29 [IQR 18–55], P = 0.02), and more unfavourable outcome (6 of 17 [35%] vs 37 of 250 [3%], P = 0.04). Compared to all other serogroups, MenW was associated with lower systolic blood pressure (103 [IQR 93–128] vs 126 [IQR 112–143], P = 0.005), lower serum thrombocytes (130 [IQR 101–171] vs 185 [IQR 145–234], P = 0.011), more systemic complications (7 of 15 [47%] vs 44 of 248 [18%], P = 0.01), and higher unfavourable outcome (6 of 16 [38%] vs 37 of 251 [15%], P = 0.03) and case fatality rates (4 of 16 [25%] vs 6 of 251 [2%], P = 0.001). Seventeen episodes of meningitis caused by cc11 were included in the prospective nationwide cohort study. Compared to other clonal complexes, cc11 was associated with higher serum C-reactive protein levels (301 [IQR 215–440] vs 214 [IQR 128–287], P = 0.007; Supplementary Table S5), and was also associated with higher rates of circulatory shock (8 of 16 [50%] vs 10 of 167 [6%], P < 0.001), unfavourable outcome (6 of 17 [35%] vs 25 of 175 [14%], P < 0.04) and death (4 of 17 [24%] vs 5 of 175 [3%], P = 0.004).

## Discussion

Our study shows that community-acquired meningococcal meningitis is currently an uncommon disease in adulthood in the Netherlands. In the period 2016–2018 there was a temporal increase in incidence up to 0.30 per 100,000 adults per year driven by increased incidence of MenW disease. In 2018, this prompted the start of a mass vaccination campaign with the MenACWY vaccine for 14- to 18-year-old Dutch children, which led to a rapid decline in MenW cases.^12^ This effect is in line with a studies from Chile and the United Kingdom that showed rapid decrease of MenW cases after implementation of the MenACWY vaccine in the nationwide vaccination programme.^25^^,^^26^ A decrease in MenW cases was also in reported in Argentina, although the incidence was reported to decrease two years prior to the implementation of routine vaccination.^27^ During the COVID-19 pandemic, non-pharmacological intervention measures were implemented in March 2020, which were associated with reduction of many infectious diseases.^28^, ^29^, ^30^ Potentially, a rebound of (meningococcal) meningitis may occur in the Netherlands, as resurgence of respiratory infections has occurred after relaxation of restriction measures against COVID-19.^31^

In our previous study from 1998 until 2002, an annual incidence of 1 per 100,000 adults per year was calculated.^8^ This is substantially higher than in our current study, with incidences ranging from 0.05–0.33 per year, which is caused by implemented immunization for MenC and a natural decline of MenB episodes. We showed that in the current study, approximately 40% were not included in the clinical cohort study, which may also be the case for our 1998–2002 cohort, resulting in an even larger decrease in incidence over the years. Clinical characteristics and outcome remained largely similar over the years.^4^ Our study, involving a subset of patients also from the current cohort, showed no statistically significant effect of dexamethasone on outcome but also no harm.^4^ In the present study with a larger dataset, we found a protective trend against unfavourable outcome (OR 0.52) but this was also not statistically significant. Furthermore, this has to be interpreted with caution as we did not correct for confounders. Nevertheless, we would advise to continue dexamethasone if *N. meningitidis* is identified as causative pathogen in a patients with bacterial meningitis, similarly to pneumococcal meningitis.

Meningococcal meningitis caused by MenW was associated with high rates of sepsis-associated features, such as lower systolic blood pressure, lower serum thrombocytes, systemic complications. These clinical features likely contributed to the high rates of unfavourable outcome (38%) and death (25%). This is in line with previous studies reporting sepsis, (epi)glottitis, gastro-intestinal symptoms, and septic arthritis in MenW disease, rather than the clinical syndrome of meningitis to be associated with MenW infection.^32^, ^33^, ^34^, ^35^, ^36^ Previously reported case-fatality rates of invasive MenW disease varied between 0 and 30%.^33^^,^^35^, ^36^, ^37^ In the time-frame of our dataset, a capsular switch of cc11 occurred from MenC to MenW, a well-known phenomenon.^38^^,^^39^ In our previous study, clonal complex 11 was only associated with serogroup C and also associated with unfavourable outcome, which might indicate that clonal complex 11 has more impact on outcome than the serogroup.^8^ Due to high collinearity between serogroup and clonal complex - almost all serogroup W and C were cc11 - the independent effect of serogroup and clonal complex could not be reliably estimated with multivariate regression analysis. Indeed, clonal complex 11 was associated with unfavourable outcome, but also age, low admission Glasgow Coma Scale scores, focal cerebral deficits on admission, and tachycardia and positive blood cultures.

In the present study, 71% of MenB isolates would theoretically be covered by the 4CMenB vaccine. As the majority episodes were caused by MenB, this may have a big impact on the overall incidence of meningococcal meningitis. Our predicted coverage is in line with previous studies reporting overall predicted strain coverage rates of 69–78% by the 4CMenB vaccine, and adults being on the lower end of the range.^40^, ^41^, ^42^, ^43^ However, most studies used genetic Meningococcal Antigen Typing System (gMATS). The gMATS excludes the NadA antigen, and considers a number of different alleles as covered or not covered, which may cause slightly higher or lower predicted coverage.^23^^,^^41^ A number of countries, including the United Kingdom, Ireland, Italy, and Australia already included the vaccine in the nationwide programme.^44^ A recent review showed that in fully vaccinated cohorts, vaccine effectiveness ranged from 50% to 100%.^45^ Herd immunity effects for this vaccine likely are limited.^46^^,^^47^ In the Netherlands, the vaccine costs are only reimbursed by health insurance for patients with specific immunodeficiencies, such as complement deficiencies, after cost-effectiveness analyses. However, as potential capsule switching of clonal complex 11 may cause MenB to have a more unfavourable outcome in the future, the potential coverage of this vaccine may be of significance. Interestingly, we showed that MenW is also often covered by the vaccine, which is in line with previous studies.^16^^,^^41^ As the polysaccharide MenACWY vaccine is included in the infant immunisation programme in the Netherlands, the clinical relevance of a MenB vaccine to protect from MenW is limited.

There are several limitations to this study. First, the NRLBM receives approximately 90% of CSF samples of all patients with positive CSF cultures in the Netherlands. Although this is a high inclusion rate, about 10% of patients with positive CSF cultures are not included, which may lead to inclusion bias.^19^ Second, the notification through the NRLBM with positive cultures will lead to an underestimation of those with negative cultures. It has been estimated that negative cultures occur in about 10–20% of patients with bacterial meningitis. This also may lead to bias, which will probably lead to underestimation of disease severity in our study because patients who are unstable or have other contraindications for immediate lumbar puncture will likely be underrepresented in our study.^48^^,^^49^ We previously showed that the number of episodes reported to the NRLBM is comparable to that reported by the Dutch National Institute for Public Health and the Environment – with compulsory notification for meningococcal disease.^2^ However, 38% of the identified episodes by the NRLBM were not included in our prospective nationwide cohort because they were either detected by CSF PCR only, or included in the first year of our cohort study while approval of medical ethics committees was still pending in a number of hospitals. Age, sex, serogroups and clonal complexes were comparable between episodes included, and not included in the nationwide cohort study.

In conclusion, adult community-acquired meningococcal meningitis has become a rare disease in the Netherlands. Between 2016 and 2018, we observed an increase in incidence of adult meningococcal meningitis, driven by increased incidence of disease caused by serogroup W, which was associated with high rates of unfavourable outcome and death. Following the mass vaccination and catch-up campaigns with the conjugate MenACWY vaccine for 14- to 18-year-old children, the incidence decreased, highlighting the need of careful and continuous surveillance of this disease. Furthermore, restrictive lockdown measures due to COVID-19 may have caused a large decrease in meningococcal meningitis cases in 2020–2021 and we may expect an increase in cases as personal contact has now normalised.

## Contributors

TMvS contributed to data gathering, data analyses, data interpretation and writing the first draft of the manuscript. NC contributed to data gathering, review, and critique of the report. NMvS contributed to data gathering and supplying microbiological data and WGS for *N. meningitis* isolates, review, and critique of the manuscript. MWB contributed to study design, data interpretation, review, and critique of the manuscript. MCB contributed to study design, data gathering, data interpretation, review, and critique of the report. DvdB contributed to study design, data interpretation, review, and critique of the report. TMvS and NC have verified the underlying data. All authors had full access to the data and accept responsibility to submit for publication.

## Data sharing statement

Data protection regulations in the Netherlands do not allow sharing of individual participant data. Data sets with selected aggregated data will be shared upon request. Proposals should be directed to prof. dr. D. van de Beek.

## Declaration of interests

N.M. van Sorge receives consultancy fees from MSD, and GSK (fees paid to Amsterdam UMC); In addition, N.M. van Sorge has a patent WO 2013/020,090 A3 (inventors: N.M. van Sorge/V. Nizet) outside the submitted work with royalties paid to University of California San Diego. Other authors: no conflicts of interest.
