# Supplementary material for: Epidemiology, clinical features and outcome of adults with meningococcal meningitis: a 15-year prospective nationwide cohort study
Source: Lancet Reg Health Eur. 2023 Apr 28;30:100640. doi: 10.1016/j.lanepe.2023.100640 (PMC10173179; doi:10.1016/j.lanepe.2023.100640)

**SUPPLEMENTARY TABLES**

**Supplementary Table S1. Clonal complexes and serogroups**

| **Characteristic** | **Overall** | **Serogroup B** | **Serogroup C** | **Serogroup W** | **Serogroup X** | **Serogroup Y** | **Non-groupable** |
| --- | --- | --- | --- | --- | --- | --- | --- |
| Total |  | 328/428 (77%) | 27/428 (6%) | 33/428 (8%) | 2/428 (1%) | 35/428 (8%) | 3/428 (1%) |
| Clonal complex |  |  |  |  |  |  |  |
| cc41/44 | 66/237 (28%) | 65/178 (37%) | 1/15 (7%) | 0/19 (0%) | 0/2 (0%) | 0/21 (0%) | 0/2 (0%) |
| cc32 | 57/237 (24%) | 57/178 (32%) | 0/15 (0%) | 0/19 (0%) | 0/2 (0%) | 0/21 (0%) | 0/2 (0%) |
| cc11 | 23/237 (10%) | 0/178 (0%) | 9/15 (60%) | 14/19 (74%) | 0/2 (0%) | 0/21 (0%) | 0/2 (0%) |
| cc269 | 21/237 (9%) | 21/178 (12%) | 0/15 (0%) | 0/19 (0%) | 0/2 (0%) | 0/21 (0%) | 0/2 (0%) |
| cc213 | 16/237 (7%) | 16/178 (9%) | 0/15 (0%) | 0/19 (0%) | 0/2 (0%) | 0/21 (0%) | 0/2 (0%) |
| cc23 | 14/237 (6%) | 0/178 (0%) | 0/15 (0%) | 0/19 (0%) | 0/2 (0%) | 13/21 (62%) | 1/2 (50%) |
| cc22 | 7/237 (3%) | 1/178 (1%) | 0/15 (0%) | 5/19 (26%) | 0/2 (0%) | 1/21 (5%) | 0/2 (0%) |
| Other | 33/237 (14%) | 18/178 (10%) | 5/15 (33%) | 0/19 (0%) | 2/2 (100%) | 7/21 (33%) | 1/2 (50%) |
| Not sequenced | 191 | 150 | 12 | 14 | 0 | 14 | 1 |

Data as n/N (%).

**Supplementary Table S2. Demographics, meningococcal serogroups and clonal complexes, compared between episodes which were and were not included in the MeninGene study**

| **Characteristic** | **Included in MeninGene, N = 274** | **Not included in MeninGene, N = 168** | **p-value** |
| --- | --- | --- | --- |
| Age | 33 (19-55) | 29 (18-57) | 0.20 |
| Sex | 142/274 (52%) | 84/166 (51%) | 0.80 |
| Serogroup |  |  | 0.48 |
| B | 206/267 (77%) | 122/161 (76%) |  |
| C | 17/267 (6.4%) | 10/161 (6.2%) |  |
| W | 16/267 (6.0%) | 17/161 (11%) |  |
| X | 1/267 (0.4%) | 1/161 (0.6%) |  |
| Y | 25/267 (9.4%) | 10/161 (6.2%) |  |
| Ungroupable | 2/267 (0.7%) | 1/161 (0.6%) |  |
| Clonal complex |  |  | 0.81 |
| cc11 | 17/192 (8.9%) | 6/45 (13%) |  |
| cc213 | 13/192 (6.8%) | 3/45 (6.7%) |  |
| cc22 | 7/192 (3.6%) | 0/45 (0%) |  |
| cc23 | 11/192 (5.7%) | 3/45 (6.7%) |  |
| cc269 | 16/192 (8.3%) | 5/45 (11%) |  |
| cc32 | 49/192 (26%) | 8/45 (18%) |  |
| cc41/44 | 52/192 (27%) | 14/45 (31%) |  |
| Other | 27/192 (14%) | 6/45 (13%) |  |

Data as n/N (%) or median (interquartile range)

**Supplementary Table S3. Sepsis-related features associated with low CSF leukocyte counts**

|  | CSF leukocyte category | | | |
| --- | --- | --- | --- | --- |
| Characteristic | <100 (N = 18) | 100-999 (N = 20) | 1000-10000 (N = 138) | >10000 (N = 78) |
| CRP - mg/L | 134 (126-174) | 140 (75-198) | 223 (126-293) | 268 (183-333)** |
| Systolic blood pressure - mmHg | 112 (98-120) | 112 (105-128) | 129 (115-144)** | 126 (116-143)** |
| Thrombocyte count - x10^9/L | 144 (96-162) | 178 (121-238)* | 198 (150-243)*** | 184 (139-216)* |
| Positive blood culture | 15/18 (83%) | 9/16 (56%) | 59/117 (50%) | 33/67 (49%) |

Values displayed as median (IQR) or n/N (%). CRP = C-reactive Protein.
* = P-value <0.05 compared to <100 CSF leukocyte group using linear regression;
** = P-value <0.01 compared to <100 CSF leukocyte group using linear regression;
*** = P-value <0.001 compared to <100 CSF leukocyte group using linear regression. No significant findings for positive blood cultures using the Fisher’s exact test (P=0.054).

**Supplementary Table S4. Clinical characteristics of patients infected with indicated meningococcal serogroup**

| **Characteristic** | **B (N = 328)^a^** | **C (N = 27)^b^** | **W (N = 33)^c^** | **Y (N = 35)^d^** | **Other (N = 5)**^e^ |
| --- | --- | --- | --- | --- | --- |
| **Demographics** |  |  |  |  |  |
| Age – years^f^ | 25 (18-54)** | 46 (31-62)* | 36 (20-59) | 49 (24-66)* | 46 (27-52) |
| Female sex | 171/326 (52%) | 13/27 (48%) | 15/33 (45%) | 17/35 (49%) | 1/5 (20%) |
| Immunocompromised | 19/206 (9%)** | 4/17 (24%) | 3/16 (19%) | 8/25 (32%)* | 1/3 (33%) |
| Diabetes Mellitus | 13/202 (6%) | 0/17 (0%) | 3/16 (19%) | 4/25 (16%) | 0/3 (0%) |
| HIV | 1/205 (0%) | 1/17 (6%) | 0/16 (0%) | 1/25 (4%) | 0/3 (0%) |
| Immunosuppressive medication | 1/205 (0%)* | 1/16 (6%) | 0/16 (0%) | 3/25 (12%)* | 0/3 (0%) |
| Alcoholism | 6/206 (3%) | 1/17 (6%) | 0/16 (0%) | 0/25 (0%) | 1/3 (33%) |
| Active Cancer | 0/205 (0%) | 1/17 (6%) | 0/16 (0%) | 0/25 (0%) | 0/3 (0%) |
| Extrameningeal infection | 16/204 (8%) | 0/16 (0%) | 1/15 (7%) | 2/25 (8%) | 0/3 (0%) |
| Pneumonia | 6/201 (3%) | 0/16 (0%) | 1/15 (7%) | 0/24 (0%) | 0/3 (0%) |
| Otitis or sinusitis | 10/196 (5%) | 0/15 (0%) | 0/13 (0%) | 2/25 (8%) | 0/3 (0%) |
| Antibiotics before admission | 4/200 (2%) | 2/16 (12%) | 0/15 (0%) | 2/24 (8%) | 0/3 (0%) |
| Duration of symptoms < 24 hours | 97/199 (49%) | 6/16 (38%) | 6/15 (40%) | 12/25 (48%) | 2/3 (67%) |
| **Presenting features** |  |  |  |  |  |
| Headache | 175/190 (92%) | 13/15 (87%) | 12/13 (92%) | 20/23 (87%) | 3/3 (100%) |
| Nausea | 137/183 (75%) | 5/13 (38%)* | 13/14 (93%) | 13/22 (59%) | 3/3 (100%) |
| Neck stiffness | 148/191 (77%) | 11/14 (79%) | 9/13 (69%) | 17/24 (71%) | 2/3 (67%) |
| Rash | 100/195 (51%)* | 6/16 (38%) | 4/14 (29%) | 5/23 (22%)* | 2/3 (67%) |
| Temperature - °C^g^ | 38.1 (37.1-39.0) | 38.7 (37.2-39.0) | 37.5 (36.8-38.5) | 38.1 (36.8-38.8) | 37.7 (37.6-38.7) |
| >38.0 °C | 106/198 (54%) | 9/16 (56%) | 6/16 (38%) | 13/25 (52%) | 1/3 (33%) |
| Heart rate - beats/min^h^ | 90 (76-103) | 84 (80-101) | 99 (84-114) | 90 (80-100) | 106 (100-108) |
| Systolic blood pressure – mmHg^i^ | 126 (113-143) | 130 (117-142) | 103 (93-128)* | 129 (110-148) | 120 (115-121) |
| Diastolic blood pressure – mmHg^j^ | 72 (61-82) | 72 (62-81) | 68 (60-84) | 74 (65-82) | 70 (70-75) |
| GCS score^k^ | 14 (11-15)* | 12 (10-14) | 13 (10-15) | 12 (10-14) | 15 (15-15) |
| Altered mental status (<14) | 85/205 (41%) | 9/16 (56%) | 9/16 (56%) | 15/25 (60%) | 0/3 (0%) |
| Coma (<8) | 19/205 (9%) | 1/16 (6%) | 2/16 (12%) | 1/25 (4%) | 0/3 (0%) |
| Seizures | 7/198 (4%) | 0/15 (0%) | 0/16 (0%) | 1/25 (4%) | 0/3 (0%) |
| Cranial nerve palsy | 6/182 (3%) | 0/14 (0%) | 0/13 (0%) | 0/23 (0%) | 0/3 (0%) |
| Aphasia, mono- or hemiparesis | 21/195 (11%) | 1/16 (6%) | 1/14 (7%) | 4/24 (17%) | 0/3 (0%) |
| Triad | 38/198 (19%) | 4/16 (25%) | 3/15 (20%) | 8/24 (33%) | 0/3 (0%) |
| **Blood results** |  |  |  |  |  |
| Thrombocytes - x10^9/L^l^ | 189 (149-234)* | 173 (130-266) | 130 (101-171)* | 167 (115-212)* | 198 (169-228) |
| Leukocytes - x10^9/L^m^ | 19 (15-24)* | 21 (11-24) | 15 (12-20) | 14 (11-22)* | 21 (21-23) |
| C-reactive protein - mg/L^n^ | 220 (129-314) | 226 (194-302) | 253 (190-368) | 231 (141-283) | 300 (278-321) |
| **CSF results** |  |  |  |  |  |
| Leukocytes - cells/mm3^o^ | 5,938 (2,182-13,053) | 5,220 (1,578-10,365) | 2,230 (1,165-7,940) | 5,332 (1,498-9,867) | 3,529 (1,786-6,198) |
| 0-99 | 13/192 (7%) | 2/16 (12%) | 1/13 (8%) | 1/23 (4%) | 1/3 (33%) |
| 100-999 | 16/192 (8%) | 1/16 (6%) | 2/13 (15%) | 1/23 (4%) | 0/3 (0%) |
| >999 | 163/192 (85%) | 13/16 (81%) | 10/13 (77%) | 21/23 (91%) | 2/3 (67%) |
| CSF:blood glucose ratio^p^ | 0.09 (0.01-0.32) | 0.01 (0.01-0.02) | 0.19 (0.01-0.34) | 0.14 (0.02-0.32) | 0.54 (0.50-0.58)* |
| CSF protein - g/L^q^ | 3.78 (1.88-6.09) | 5.30 (3.49-6.80) | 3.90 (3.01-6.65) | 5.15 (2.65-6.85) | 0.63 (0.51-0.76)* |
| Opening pressure - cm H2O^r^ | 41 (30-50) | 38 (36-42) | 36 (24-50) | 47 (42-48) | 29 (24-32) |
| **Microbiology** |  |  |  |  |  |
| Positive CSF culture | 176/206 (85%) | 15/17 (88%) | 14/16 (88%) | 20/25 (80%) | 3/3 (100%) |
| Positive blood culture | 74/163 (45%)* | 7/13 (54%) | 11/13 (85%)* | 17/23 (74%)* | 1/3 (33%) |
| Positive Gram stain | 123/162 (76%) | 13/13 (100%)* | 9/10 (90%) | 12/19 (63%) | 1/2 (50%) |
| **Complications** |  |  |  |  |  |
| Pneumonia | 9/195 (5%) | 0/16 (0%) | 0/13 (0%) | 0/24 (0%) | 0/3 (0%) |
| Arthritis | 7/200 (4%) | 1/16 (6%) | 0/13 (0%) | 2/25 (8%) | 0/3 (0%) |
| Circulatory shock | 12/198 (6%)* | 3/16 (19%) | 6/15 (40%)** | 2/23 (9%) | 0/3 (0%) |
| Respiratory failure | 17/202 (8%) | 2/16 (12%) | 3/15 (20%) | 2/25 (8%) | 0/3 (0%) |
| Mechanical ventilation | 34/203 (17%) | 4/15 (27%) | 3/15 (20%) | 2/25 (8%) | 0/3 (0%) |
| Focal neurological deficits | 24/197 (12%) | 2/15 (13%) | 2/12 (17%) | 4/25 (16%) | 0/3 (0%) |
| Seizures | 2/199 (1%)* | 1/15 (7%) | 1/15 (7%) | 2/23 (9%) | 0/3 (0%) |
| Cerebral infarction | 4/198 (2%) | 1/15 (7%) | 0/14 (0%) | 1/24 (4%) | 0/3 (0%) |
| **Score on Glasgow Outcome Scale** |  |  |  |  |  |
| 1 (death) | 4/206 (2%)* | 2/17 (12%) | 4/16 (25%)** | 0/25 (0%) | 0/3 (0%) |
| 2 (vegetative state) | 0/206 (0%) | 0/17 (0%) | 0/16 (0%) | 0/25 (0%) | 0/3 (0%) |
| 3 (severe disability) | 3/206 (1%) | 2/17 (12%) | 1/16 (6%) | 0/25 (0%) | 0/3 (0%) |
| 4 (moderate disability) | 20/206 (10%) | 2/17 (12%) | 1/16 (6%) | 4/25 (16%) | 0/3 (0%) |
| 5 (mild or no disability) | 179/206 (87%) | 11/17 (65%)* | 10/16 (62%)* | 21/25 (84%) | 3/3 (100%) |
| Focal neurological deficits at discharge | 15/177 (8%) | 1/13 (8%) | 0/9 (0%) | 1/21 (5%) | 0/3 (0%) |

Data as n/N (%) or median (interquartile range). ^a^206 included in the MeninGene study  ^b^17 included in the MeninGene study ^c^16 included in the MeninGene study ^d^25 included in the MeninGene study ^e^Including serogroup X (n=2) and non-groupable strains (n=3), 3 included in the MeninGene study. ^f^Was known for 328 MenB episodes, 27 MenC episodes, 33 MenW episodes, 35 MenY episodes and 5 episodes in the other category. ^g^Was known for 198 MenB episodes, 16 MenC episodes, 16 MenW episodes, 25 MenY episodes and 3 episodes in the other category. ^h^Was known for 196 MenB episodes, 16 MenC episodes, 15 MenW episodes, 25 MenY episodes and 3 episodes in the other category. ^i^Was known for 198 MenB episodes, 16 MenC episodes, 15 MenW episodes, 25 MenY episodes and 3 episodes in the other category. ^j^Was known for 198 MenB episodes, 16 MenC episodes, 15 MenW episodes, 25 MenY episodes and 3 episodes in the other category. ^k^Was known for 206 MenB episodes, 16 MenC episodes, 16 MenW episodes, 25 MenY episodes and 3 episodes in the other category. ^l^Was known for 194 MenB episodes, 14 MenC episodes, 13 MenW episodes, 25 MenY episodes and 2 episodes in the other category. ^m^Was known for 205 MenB episodes, 14 MenC episodes, 14 MenW episodes, 25 MenY episodes and 3 episodes in the other category. ^n^Was known for 198 MenB episodes, 13 MenC episodes, 14 MenW episodes, 25 MenY episodes and 2 episodes in the other category. ^o^Was known for 192 MenB episodes, 16 MenC episodes, 13 MenW episodes, 23 MenY episodes and 3 episodes in the other category. ^p^Was known for 188 MenB episodes, 11 MenC episodes, 14 MenW episodes, 24 MenY episodes and 2 episodes in the other category. ^q^Was known for 196 MenB episodes, 15 MenC episodes, 15 MenW episodes, 23 MenY episodes and 2 episodes in the other category. ^q^Was known for 92 MenB episodes, 7 MenC episodes, 5 MenW episodes, 4 MenY episodes and 3 episodes in the other category. * <0.05, **<0.005 compared to all other serogroups.

**Supplementary Table S5. Clinical characteristics of patients with meningitis caused by meningococci of clonal complex 11 vs other clonal complexes**

| **Characteristic** | **Non-cc11, N = 175** | **Cc11, N = 17** | **p-value** |
| --- | --- | --- | --- |
| **Demographics** |  |  |  |
| Age – years^a^ | 29 (19-54) | 49 (26-57) | 0.10 |
| Female sex | 85/175 (49%) | 9/17 (53%) | 0.80 |
| Immunocompromised | 26/175 (15%) | 2/17 (12%) | >0.99 |
| Diabetes Mellitus | 16/173 (9%) | 1/17 (6%) | >0.99 |
| HIV | 1/174 (1%) | 1/17 (6%) | 0.17 |
| Immunosuppressive medication | 3/174 (2%) | 0/17 (0%) | >0.99 |
| Alcoholism | 6/175 (3%) | 0/17 (0%) | >0.99 |
| Active Cancer | 1/174 (1%) | 0/17 (0%) | 0.60 |
| Extrameningeal infection | 13/173 (8%) | 0/16 (0%) | 0.61 |
| Pneumonia | 6/172 (3%) | 0/16 (0%) | >0.99 |
| Otitis or sinusitis | 7/168 (4%) | 0/14 (0%) | >0.99 |
| Antibiotics before admission | 4/169 (2%) | 2/17 (12%) | 0.095 |
| Duration of symptoms < 24 hours | 85/170 (50%) | 6/17 (35%) | 0.31 |
| **Presenting features** |  |  |  |
| Headache | 146/160 (91%) | 14/16 (88%) | 0.64 |
| Nausea | 114/155 (74%) | 14/17 (82%) | 0.57 |
| Neck stiffness | 123/162 (76%) | 10/14 (71%) | 0.75 |
| Rash | 76/163 (47%) | 4/16 (25%) | 0.12 |
| Temperature - °C^b^ | 38.2 (37.2-39.0) | 38.0 (37.0-39.0) | 0.72 |
| >38.0°C | 96/169 (57%) | 8/16 (50%) | 0.61 |
| Heart rate - beats/min^c^ | 92 (80-103) | 99 (84-116) | 0.15 |
| Systolic blood pressure – mmHg^d^ | 126 (113-142) | 120 (96-135) | 0.075 |
| Diastolic blood pressure – mmHg^e^ | 72 (65-82) | 65 (62-78) | 0.27 |
| GCS score^f^ | 14 (11-15) | 14 (12-15) | 0.54 |
| Altered mental status (<14) | 74/175 (42%) | 8/16 (50%) | 0.60 |
| Coma (<8) | 14/175 (8%) | 2/16 (12%) | 0.63 |
| Seizures | 6/170 (4%) | 0/17 (0%) | >0.99 |
| Cranial nerve palsy | 3/153 (2%) | 0/15 (0%) | >0.99 |
| Aphasia, mono- or hemiparesis | 15/162 (9%) | 1/16 (6%) | >0.99 |
| Triad | 33/167 (20%) | 5/17 (29%) | 0.35 |
| **Blood results** |  |  |  |
| Thrombocytes - x10^9/L^g^ | 180 (144-230) | 130 (94-221) | 0.069 |
| Leukocytes - x10^9/L^h^ | 19 (14-23) | 15 (10-22) | 0.12 |
| C-reactive protein - mg/L^i^ | 204 (128-287) | 301 (215-440) | 0.007 |
| **CSF results** |  |  |  |
| Leukocytes - cells/mm^3,j^ | 5,100 (1,590-11,341) | 3,203 (317-7,776) | 0.36 |
| 0-99 | 12/165 (7%) | 3/14 (21%) |  |
| 100-999 | 14/165 (8%) | 2/14 (14%) |  |
| >999 | 139/165 (84%) | 9/14 (64%) |  |
| CSF:blood glucose ratio^k^ | 0.12 (0.01-0.40) | 0.02 (0.01-0.26) | 0.62 |
| CSF protein - g/L^l^ | 3.57 (1.68-6.00) | 4.69 (3.16-6.58) | 0.26 |
| Opening pressure - cm H2O^m^ | 38 (28-50) | 37 (27-47) | 0.85 |
| **Microbiology** |  |  |  |
| Positive CSF culture | 165/175 (94%) | 17/17 (100%) | 0.60 |
| Positive blood culture | 78/146 (53%) | 10/15 (67%) | 0.42 |
| Positive Gram stain | 114/141 (81%) | 12/12 (100%) | 0.13 |
| **Complications** |  |  |  |
| Pneumonia | 8/162 (5%) | 0/15 (0%) | >0.99 |
| Arthritis | 6/168 (4%) | 1/15 (7%) | 0.46 |
| Circulatory shock | 10/167 (6%) | 8/16 (50%) | <0.001 |
| Respiratory failure | 17/170 (10%) | 4/16 (25%) | 0.088 |
| Mechanical ventilation | 29/170 (17%) | 5/16 (31%) | 0.18 |
| Focal neurological deficits | 18/166 (11%) | 3/13 (23%) | 0.18 |
| Seizures | 3/166 (2%) | 2/16 (12%) | 0.062 |
| Cerebral infarction | 4/167 (2%) | 1/15 (7%) | 0.35 |
| **Score on Glasgow Outcome Scale** |  |  | 0.010 |
| 1 (death) | 5/175 (3%) | 4/17 (24%) |  |
| 2 (vegetative state) | 0/0 (0%) | 0/0 (0%) |  |
| 3 (severe disability) | 2/175 (1%) | 0/17 (0%) |  |
| 4 (moderate disability) | 18/175 (10%) | 2/17 (12%) |  |
| 5 (mild or no disability) | 150/175 (86%) | 11/17 (65%) |  |
| Focal neurological deficits at discharge | 11/144 (8%) | 0/13 (0%) | 0.60 |

Data as n/N (%) or median (IQR). ^a^Was known in x cc11 episodes and x non-cc11 episodes. ^b^Was known in 16 cc11 episodes and 169 non-cc11 episodes. ^c^Was known in 15 cc11 episodes and 165 non-cc11 episodes. ^d^Was known in 15 cc11 episodes and 167 non-cc11 episodes. ^e^Was known in 15 cc11 episodes and 167 non-cc11 episodes. ^f^Was known in 16 cc11 episodes and 175 non-cc11 episodes. ^g^Was known in 14 cc11 episodes and 164 non-cc11 episodes. ^h^Was known in 15 cc11 episodes and 173 non-cc11 episodes. ^i^Was known in 14 cc11 episodes and 166 non-cc11 episodes. ^j^Was known in 14 cc11 episodes and 165 non-cc11 episodes. ^k^Was known in 13 cc11 episodes and 158 non-cc11 episodes. ^l^Was known in 15 cc11 episodes and162 non-cc11 episodes. ^m^Was known in 6 cc11 episodes and 67 non-cc11 episodes.

**SUPPLEMENTARY FIGURES**

**Supplementary figure S1. Incidence of meningococcal meningitis per clonal complex**


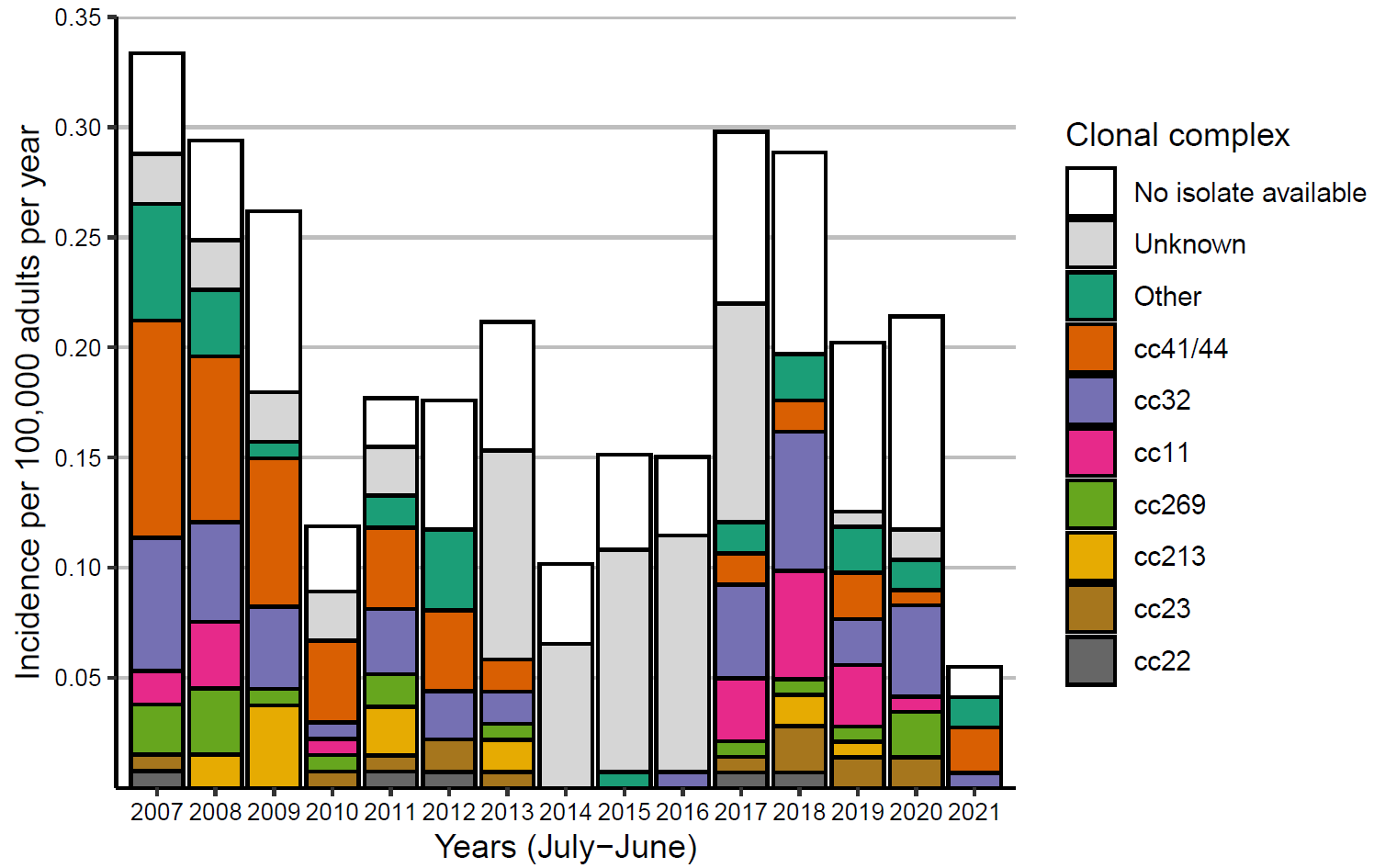

Supplement: Supplementary Fig. S1 and Tables S1–S5 [file mmc1.docx]
